# The sphenoid sinus, foramen rotundum and vidian canal: a radiological study of anatomical relationships^☆^

**DOI:** 10.1016/j.bjorl.2016.04.013

**Published:** 2016-05-24

**Authors:** Alireza Mohebbi, Shahin Rajaeih, Mahdi Safdarian, Parisa Omidian

**Affiliations:** Iran University of Medical Sciences, Department of Otolaryngology, Head and Neck Surgery, Tehran, Iran

**Keywords:** Foramen rutundum, Sphenoid sinus, Vidian canal, Forame redondo, Seio esfenoidal, Canal pterigoideo

## Abstract

**Introduction:**

The sphenoid sinus is an important structure in ventral skull base surgeries that is surrounded by several vital anatomical structures including the internal carotid arteries, optic nerve and cranial nerves inside the cavernous sinus. In addition, the foramen rotundum is a small canal deeply situated in the base of the skull, which represents the way for exit of the maxillary nerve. Understanding of the sphenoid bone anatomical relationships is central to the expanded endonasal approaches to the skull base.

**Objective:**

To record and analyze the measurement indexes of the sphenoid sinus and foramen rotundum in the coronal plane of normal computer tomography scans.

**Methods:**

Patients underwent paranasal sinuses computer tomography scan from June 2014 to November 2015 were retrospectively entered this cross-sectional study. We obtained several morphometric measurements from both the right and left sides using computer software. We also classified foramen rotundum and vidian canal types and determined position of the foramen rotundum regarding to base of lateral pterygoid plate.

**Results:**

One-hundred patients with the mean age of 38.56 ± 18.51 years entered this study. Mean bilateral FR distances were 38.48 ± 3.87 mm. Average right and left FRs distances to midline were 19.00 ± 2.07 and 19.34 ± 2.17 mm, respectively (*p* = 0.03). Twenty-eight cases (28%) had type I vidian canal, 48% and 24% had type II and III vidian canals, respectively. Four patients (4%) had type I rotundum foramen, 28% and 44% had type IIa and IIb, respectively and 24% had type III rotundum foramen. The position of foramen rotundums regarding to the base of lateral pterygoid plate was online in 50% of cases, medially placed in 47% and laterally placed in 3% of cases.

**Conclusion:**

The results of this study can be used to provide a better anatomical understanding of the area, which is necessary for endoscopic skull base surgeons.

## Resumo

**Introdução:**

O seio esfenoidal (SE) é uma estrutura importante em cirurgias da base do crânio, que está cercada por várias estruturas anatômicas vitais, como as artérias carótidas internas, o nervo óptico e os nervos cranianos no interior do seio cavernoso. Além disso, o forame redondo (FR) é um pequeno canal profundamente situado na base do crânio, que representa a forma de saída do nervo maxilar. Compreender as relações anatômicas do osso esfenoidal é fundamental para as abordagens endonasais expandidas da base do crânio.

**Objetivo:**

Registrar e analisar os índices de medição do SE e FR no plano coronal de exames normais de TC.

**Método:**

Os pacientes que foram submetidos a TC dos seios paranasais (SPN) de junho de 2014 a novembro 2015 foram retrospectivamente incluídos neste estudo transversal. Obtivemos várias medidas morfométricas de ambos os lados direito e esquerdo usando software de computador. Também classificamos os tipos de FR e canal pterigoideo (CP) e determinamos a posição do FR em relação à base da placa pterigoide lateral.

**Resultados:**

Cem pacientes com a média de idade de 38,56 ± 18,51 anos foram incluídos neste estudo. As distâncias médias bilaterais de FR foram de 38,48 ± 3,87 milímetros. As distâncias médias direita e esquerda dos FR até a linha média foram de 19,00 ± 2,07 e 19,34 ± 2,17 mm, respectivamente (*p* = 0,03). Vinte e oito casos (28%) tinham canal pterigoideo do tipo I, 48% e 24% canais pterigoideos de tipo II e III, respectivamente. Quatro pacientes (4%) tinham forame redondo do tipo I, 28% e 44% do tipo II-a e II-b, respectivamente, e 24% forame redondo do tipo III. A posição dos FR em relação à base da placa pterigoide lateral era em linha em 50% dos casos, medialmente posicionados em 47% e lateralmente posicionados em 3% dos casos.

**Conclusão:**

Os resultados deste estudo podem ser utilizados para promover uma melhor compreensão anatômica da área, necessária para cirurgias endoscópicas da base do crânio.

## Introduction

The sphenoid sinus (SS) is an important structure in ventral skull base surgeries that is not only the natural route for access to the sellar, parasellar, suprasellar and clival regions, but also a path of access to Meckel's cave and the middle cranial fossa. It is surrounded by several vital anatomical structures including the internal carotid arteries, optic nerve and cranial nerves inside the cavernous sinus. The surgical window to the middle cranial fossa is located in the pterygoid body of the sphenoid bone.^1^

The SS is present as a small cavity at birth, but its main development takes place after puberty. In early life, it extends posteriorly into the presellar area and subsequently expands into the area below and behind the sella turcica, reaching its full size during adolescence. As the sinus enlarges, it may partially encircle the optic canals. When the sinus is exceptionally large, it extends into the roots of the pterygoid processes or greater wing of the sphenoid bone and may even extend into the basilar part of the occipital bone. In the well-pneumatized SS, only a thin layer of bone may separate the sinus from important contiguous structures. The close proximity of these neurovascular structures with potentially very thin bony separation or even bony dehiscence contributes to the clinical importance of these anatomical relationships.

Few studies used thin-cut (1 mm) CT data to study the pneumatization of the lateral sphenoid or pterygoid recess.^2^, ^3^ The emergence of endoscopic skull base surgery as an accepted surgical modality over the past years has led to new challenges with respect to gaining a better understanding of the endonasal anatomy of the area. As such, new paradigms of anatomical relationships have evolved into instrumental landmarks to the endoscopic skull base surgeon.^4^

Alongside the past development of endoscopic sinus surgery, knowledge about the anatomy of the sinuses has become crucial for surgeons. The SS is one of the most variable of all sinuses. Its relations to vital vascular and nervous elements make its approach a challenge for endoscopic surgeons.^5^ In addition, the foramen rotundum (FR), is a small canal deeply situated in the base of the skull, which represents the way for exit of the second branch of the trigeminal nerve (maxillary nerve).^6^ Its medial border is formed by lateral wall of SS and runs downwards and laterally in an oblique path and joins the middle cranial fossa with the pterygopalatine fossa.^7^

Individualization and analyze of SS is difficult and necessitates a precise and adapted technique, as well as knowledge of its properties anatomical relationships.^6^ Its involvement which is preferentially related to tumoral pathologies (particularly with retrograde perineural invasion) profoundly modifies the prognosis of the disease and necessitate a multidisciplinary therapeutic discussion.^6^ Impressions caused by neurovascular coursing provide several important surgical landmarks for locating these vital structures and avoiding their injury.^8^ Understanding of the sphenoid bone anatomical relationships is central to the expanded endonasal approaches to the skull base.^4^

Even though computer tomography (CT) opened then era of detailed morphological studies, due to lack of sufficient and precise literature in vicinity and properties of the FR, we designed this anatomical study to evaluate normal CT scans of patients, record and analyze distances and angles. The goal of this study was to present a classification based on the measurement indexes in the coronal plane that can be used to instruct preoperative planning for endoscopic endonasal surgery.

## Methods

### Approval statement of the ethics committee

This study was approved by the local ethics committee of the ENT – Head & Neck surgery research center of Hazrat-e-Rasool Akram Hospital with the approval protocol number of 94-11860. The information of patients remained confidential and was only used for research purposes.

### Study design

**Figure 1:**
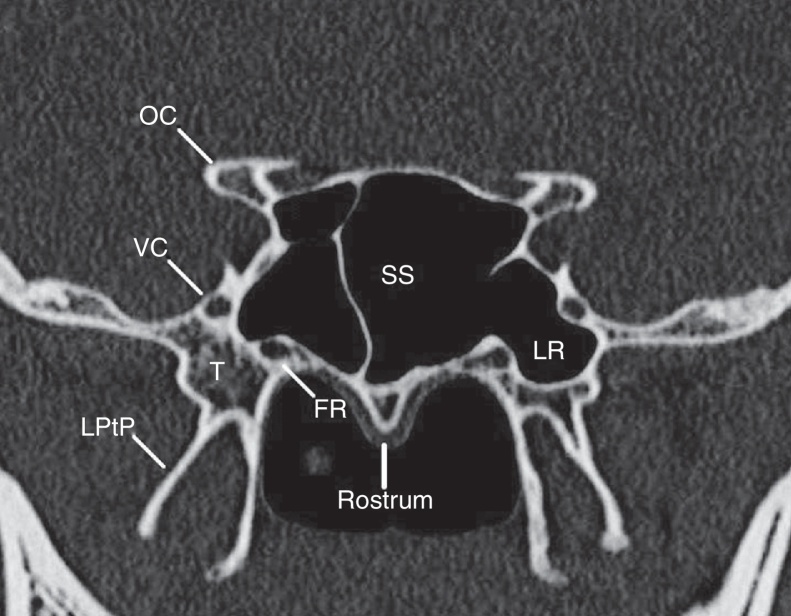
The coronal section at which both the vidian canal and foramen rotundum are visualized (usually at the midpoint of sphenoid sinus). SS, sphenoid sinus; FR, foramen rotundum; VC, vidian canal; LPtP, lateral pterygoid plate; OC, optic canal; LR, lateral recess.

This retrospective cross-sectional study was designed on adult patients who underwent paranasal sinuses (PNS) CT scan (3 mm slices thickness) for any reason, from June 2014 to November 2015 in Hazrat-e-Rasool Akram Hospital (a tertiary-care medical center) Iran University of Medical Sciences, Tehran, Iran. Exclusion criteria included individuals younger than 18 years of age or with known skull base pathology including maxillofacial fractures, sinonasal tumors or polyposis, disruption of the skull base or notable rhinosinusitis (inflammatory changes that precluded visualization of skull base anatomy). For each included patient, we obtained measurements from both the right and left sides using MacroPACS software in both axial and coronal planes. The first coronal image section at which both the VC and FR were visualized was chosen for the quantitative analysis. This section (as determined on axial and sagittal images) was usually at the midpoint of sphenoid sinus (Fig. 1).

### Measurements

**Figure 2:**
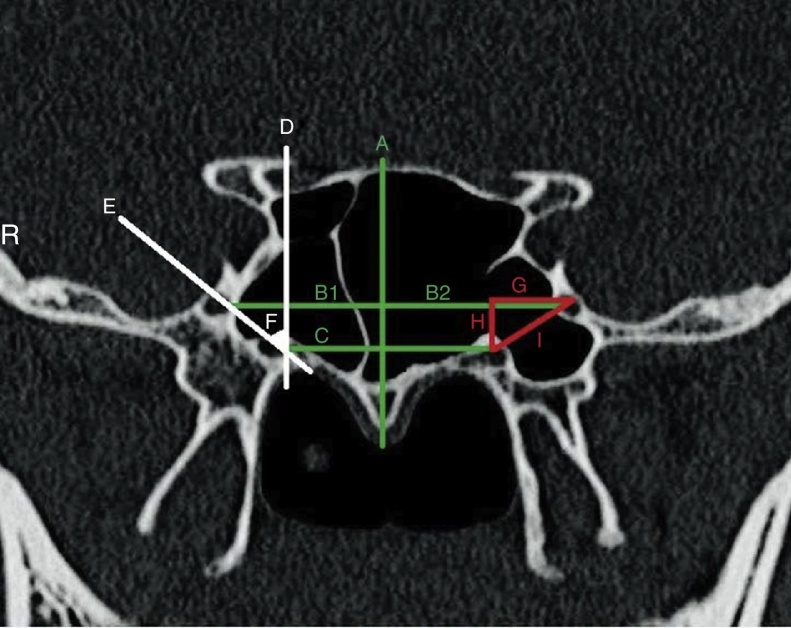
Measurement indexes of the study. A, Imaginary midline vertical to the rostrum; B (1), distance between right FR to midline; B (2), distance between left FR to midline; C, imaginary horizontal line connecting the VCs; D, imaginary vertical line passing the VC; E, imaginary line connecting FR to VC; F, the rotundum angle with the vertical line; G, H and I, horizontal, vertical and direct distances between FR and VC, respectively (R, right).

We measured several morphometric parameters according to an imaginary midline vertical to the rostrum (Fig. 2):

Distance between right and left FRs;
Distances from midline to right and left FRs;
Direct distance between the VC and the FR on each side;
Horizontal distance between the VC and the FR on each side (distances between two vertical line that intersecting the FR and the VC);
Vertical distance between the VC and the FR on each side (distance between two horizontal line that intersecting the FR and the VC).
Right and left rotundum angles (calculated as the angle between the imaginary line connecting FR to VC and vertical line passing the VC).

**Figure 3:**
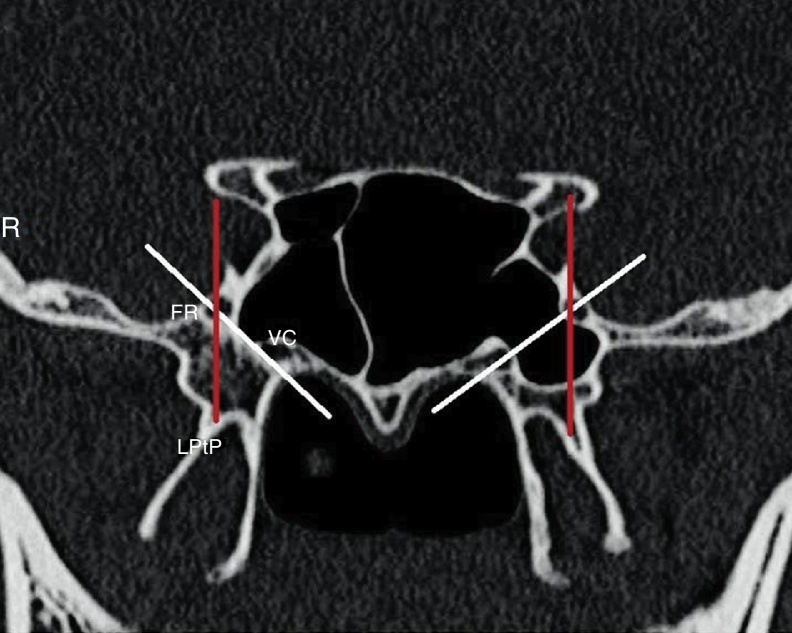
Sphenoid sinus pneumatization classification according to the imaginary line connecting foramen rotundum to vidian canal; white lines (right, lateral recess; left, tangent) and position of the FR regarding to base of lateral pterygoid plate; red lines (right and left, online) (R, right).

Position of the FR regarding to base of lateral pterygoid plate defined as (Fig. 3):

Online – when FR is tangent to the lateral pterygoid plate;
Medial – when FR is placed medially regard the lateral pterygoid plate;
Lateral – when FR is placed laterally regard the lateral pterygoid plate.

SS pneumatization was classified according to the imaginary line connecting FR to VC (Fig. 3):

Lateral recess – when the sinus is pneumatized to the lateral of the imaginary line;
Tangent – when the sinus is pneumatized tangent to the imaginary line;
Less pneumatized – when the sinus is pneumatized medial to the imaginary line.

Mid-sphenoid position was defined according to the space below the mid-sphenoid coronal section that is nasopharyngeal, choana or nasal cavity.

**Figure 4:**
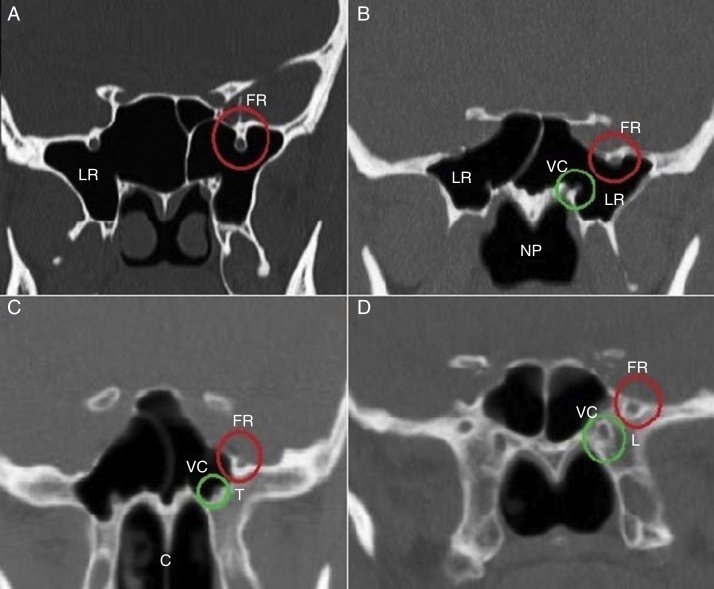
Foramen rotundum (FR), vidian canal (VC) and sphenoid sinus (SS) pneumatization classifications. A, FR type I and SS lateral recess (LR); B, FR type IIa, VC type I and SS lateral recess (LR); C, FR type IIb, VC type II and SS left side tangent (T); D, FR type III, VC type III and SS less pneumatized (L). NP, nasopharyngeal; C, choana.

Three types of FR defined as the following (Fig. 4):

Type I – when FR is placed completely within the sinus cavity;
Type IIa – when a part of FR is in the sinus cavity or partially protruding into the SS;
Type IIb – when FR is tangent to the sinus wall;
Type III – when FR is placed completely within the sphenoid bone.

The VC was also classified into three types based on CT findings (Fig. 4):

Type 1 – when VC is completely within the sphenoid sinus;
Type 2 – when VC is on the floor of the sphenoid sinus or partially protruding into the sphenoid sinus;
Type 3 – when VC is completely embedded in the sphenoid corpus.

The classification used for the types of FR is created by ourselves but the VC classification was adopted from Lee et al.^9^

### Statistical analysis

Data entered and analyzed via SPSS version 22 software (SPSS Inc, Chicago, IL, USA). Quantitative variables (including distances) expressed as mean and standard deviation (SD). The Student's *t* test and pared *T*-test were used to determine statistical significance between right and left distances. The null hypothesis assumed no difference between the groups tested. *p*-Values less than 0.05 defined as significant.

## Results

### Imaging data

A total number of one-hundred patients with the mean age of 38.56 ± 18.51 years (ranging from 18 to 86) were randomly selected from the radiographic database of the Department of Otolaryngology-Head & Neck surgery of Hazrat-e-Rasool Akram Hospital. Half of the patients (50 cases) were male. Mean bilateral FR distances were 38.48 ± 3.87 mm (range 30–48 mm). The SS pneumatization was categorized to be lateral recess in 54% of cases, tangent in 26% and less pneumatized in 20% of cases. Mid-sphenoid position was above the choana in 74% of cases, and above the nasopharyngeal and nasal cavities in 10% and 16% of cases, respectively.

Average right and left FRs distances to midline were 19.00 ± 2.07 and 19.34 ± 2.17 mm, respectively (*p* = 0.03). Average right FR to right VC distance were 5.89 ± 2.4 mm and 5.06 ± 2.03 mm, horizontally and vertically, respectively; direct distance calculated as 8.16 ± 2.27 mm. Average left FR to left VC distance were 5.93 ± 2.13 and 5.49 ± 2.13 mm, horizontally and vertically, respectively; direct distance calculated as 9.20 ± 2.15 mm. Horizontal, vertical and direct distances between the right and left FRs to VCs, had not statistically significant difference (*p* = 0.764–0.676 and *p* = 0.952, respectively) (Table 1).

**Table 1:** Measurements index of foramen rotundum distances toward midline axis, vidian canal and base of lateral pterygoid plate.

	Right (mean ± SD)	Left (mean ± SD)	*p*-Value
*Foramen rotundum to midline axis (mm)*	19.00 ± 2.07	19.34 ± 2.17	**0.03**

*Foramen rotundum to vidian canal (mm)*			
Horizontal	5.89 ± 2.4	5.93 ± 2.13	0.764
Vertical	5.06 ± 2.03	5.49 ± 2.13	0.676
Direct	8.16 ± 2.27	9.20 ± 2.15	0.952

*Rotundum angles (degree)*	46.76 ± 12.32	46.40 ± 10.67	0.647

Twenty-eight cases (28%) had type I vidian canal, 48% and 24% had type II and III vidian canals, respectively. Four patients (4%) had type I rotundum foramen, 28% and 44% had type IIa and IIb, respectively and 24% had type III rotundum foramen (Table 2).

**Table 2:** Types of vidian canal and foramen rotundum.

	I	II		III
Foramen rotundum	4	*28 (IIa)*	*44 (IIb)*	24
Vidian canal	28	48		24

The position of right FR regarding to the base of right lateral pterygoid plate was online in 48%, medially placed in 50% and laterally placed in 2% of cases. The position of left FR regarding to the base of left lateral pterygoid plate was online in 52%, medially placed in 44% and laterally placed in 4% of cases (Table 3).

**Table 3:** Right and left FRs positions in relation to base of lateral pterygoid plate.

	Right	Left	Total
Online placed	48%	52%	50%
Medially placed	50%	44%	47%
Laterally placed	2%	4%	3%

## Discussion

The results of this study provides a radiological review about the anatomical relationships of the SS and FR with the other anatomical landmarks of the area such as VC and base of lateral pterygoid plate which can take into accounts for sphenoid endoscopic and other surgical procedures of the area. We have described the radiographic anatomy of the FR in terms of distance from the midline axis and the types of FRs and VCs. In CT scans interpreted in the coronal plane, the FR was found to have asymmetrically distances from the midline axis, as shown in Table 1. This serves as a critical piece of information that the endoscopic skull base surgeon can use when attempting to safely localize the FR during approaches through the SS. In addition, the FRs distances to VCs were symmetrical in horizontal, vertical and direct axes. This finding may help the better localization of FR in relation to VC, which facilitate its safe identification and help the surgeon avoid an inadvertent injury to their anatomical integrity.

As it is shown in Table 3, we observed for the first time that almost all of the FR were placed either online or medial to the base of lateral pterygoid plate and only in 3% of all cases were placed lateral to the base of lateral pterygoid plate. In addition, the classification that showed the majority (74%) of mid sphenoid section positions were at the level of choana was for the first time presented in our study.

VC and FR classifications were the same bilaterally in most of the cases. Only 2 cases had different types of VCs and ten cases had different types of FRs on the two sides, which were classified and analyzed in different groups according to their classification. Remarkably, we found that the type of VC classification was always the same or one level behind the FR type in our patients. For example, when the VC was completely within the SS (type I), the FR was either placed completely within the sinus cavity (type I) or partially protruding into the SS (type II). When the VC was on the floor of the SS or partially protruding into the SS (type II), the FR was either partially protruding into the SS (type IIa), tangent to the sinus wall (type IIb) or placed completely within the sphenoid bone (type III). Finally, when the VC was completely embedded in the sphenoid corpus (type III), the FR was always completely within the sphenoid bone (type III). To our knowledge, this is the first report of such finding in PNS CT-scan. As we found in PNS CT scans of our patients, more than half of the SSs (54%) were pneumatized lateral to the imaginary line connecting FR to the VC.

During the past years, endonasal endoscopic surgery has gained great importance in sinus surgery and in some neurosurgical approaches. Due to this great evolution of sinus surgery, knowledge of the sinus anatomy has become crucial. The relations of the SS with structures around are close when the sinus is well pneumatized. When this happens, the surrounding vessels and nerves are seen in the sinus cavity as irregularities or ridges. The pneumatization of the sphenoid to the pterygoid processes is an extension of the sinus between the maxillary nerve and the nerve of the pterygoid canal (vidian nerve).

The most important relations of the sphenoid are on its superior and lateral walls, with the internal carotid artery and the optic nerve. These have been shown to have variable pathways alongside the sphenoid.^5^ The SSs are asymmetric cavities inside the sphenoid body separated by a bony septum. Literature describes this septum as being rarely situated on the median plane but very often deviated laterally to one side or the other. Its pathology is nowadays mostly approached trough endoscopic surgery, with some limits. Due to its location and its relations, it is modernly often used by rhinologists and neurosurgeons as a pathway to parts of the central nervous system, with new techniques being invented at a very high rate. Having a high variability, its anatomical relations and their variations have to be well understood prior to any surgical intervention.^5^

It is vital that the surgeon is informed about the variations in order to avoid vital complications during surgery. The endoscopic endonasal approaches are commonly used to access the middle skull base areas of the lateral cavernous sinus (mostly for tissue diagnosis or surgical decompression), Meckel's cave (for removal of trigeminal schwannomas or meningiomas, nerve resection margins for sinonasal malignancy with perineural invasion), and anterolateral middle fossa triangle (for repair of cerebrospinal fluid leaks and pseudomeningoceles).

The SS is one of the most morphologically variable and surgically important structures of the skull base. Located below the sella turcica, neighbored by parasellar regions, such as the orbital apex, pterygopalatine fossa and lateral sellar region (cavernous sinus), it is clinically related to these and surgically relevant as corridor for various approaches.^10^

Recently, some radiological studies have been designed in order to provide a better comprehension of this complex area and by defining anatomical variables and distances using the anatomical landmarks of the area. After studying the CT images of 100 and 18 SSs in adults and cadavers, respectively, Wang et al. proposed a new classification system for sinus extension including lateral, clival, lesser wing, anterior, and combined.^3^ Vescan et al. analyzed the relationships of VC to the internal carotid artery in 44 CT scan to describe the anatomy and relationships of the VC to known endonasal and skull base landmarks. They found that the degree of pneumatization of the SS is highly variable and reported some measurement indexes such as the mean length of the VC and the anatomical location of the VC and the anterior genu of the petrous internal carotid artery.^4^ Kasemsiri et al. to define anatomical landmarks for the preoperative planning of endoscopic endonasal transpterygoid approaches, reviewed images from high-resolution maxillofacial CT scans. They reported the average distance from midline to left FR 19.11 mm and to right FR 17.67 mm (*p* = 0.04). We also found that the average distance of midline to left FR to be significantly more than to right FR (*p* = 0.03). The average horizontal and vertical distances from FR to VC in Kasemsiri's study showed no significant difference between right and left sides, as we found the same result in our population.^11^

In another similar study, Vaezi et al. in 2015 used high-resolution CT scans to present a classification based on the degree of pneumatization of the SS in the coronal plane. They also measured the association of SS pneumatization with the location of the FR and the VC and reported that the distance separating the FR and the VC correlated strongly with the depth of the lateral recess.^1^

The aim of all of the above studies and the similar studies like ours is to give a better comprehension of the anatomical situation of this complex area in order to instruct better preoperative planning for endoscopic endonasal surgeries. As a result of these studies, an endoscopic skull base surgeon has a number of anatomical landmarks and measurements that may be helpful in safely localization of the foramen rotundum during endonasal approaches to the skull base.

## Conclusion

This paper makes a review about the anatomical relations of foramen rotundum with the other endonasal landmarks such as VC and lateral pterygoid plate. The results of this study can be used to provide a better anatomical understanding of the area, which is necessary for endoscopic skull base surgeons. In order to generalize the results of this study, epidemiological studies with larger sample sizes are recommended in addition to clinical studies for a better identification of the relationships between anatomical landmarks of the skull base.

## Conflicts of interest

The authors declare no conflicts of interest.
